# Prediction of disulfide connectivity in proteins with machine-learning methods and correlated mutations

**DOI:** 10.1186/1471-2105-14-S1-S10

**Published:** 2013-01-14

**Authors:** Castrense Savojardo, Piero Fariselli, Pier Luigi Martelli, Rita Casadio

**Affiliations:** 1 Department of Computer Science and Engineering, University of Bologna, Via Mura Anteo Zamboni 7, 41029 Bologna, Italy; 2 Biocomputing Group, University of Bologna, via Selmi 3, 40126 Bologna, Italy; 3 CIRI-Life Science and Health Technologies/Department of Biology, Via San Giacomo 9/2, 40129, Bologna, Italy

## Abstract

**Background:**

Recently, information derived by correlated mutations in proteins has regained relevance for predicting protein contacts. This is due to new forms of mutual information analysis that have been proven to be more suitable to highlight direct coupling between pairs of residues in protein structures and to the large number of protein chains that are currently available for statistical validation. It was previously discussed that disulfide bond topology in proteins is also constrained by correlated mutations.

**Results:**

In this paper we exploit information derived from a corrected mutual information analysis and from the inverse of the covariance matrix to address the problem of the prediction of the topology of disulfide bonds in Eukaryotes. Recently, we have shown that Support Vector Regression (SVR) can improve the prediction for the disulfide connectivity patterns. Here we show that the inclusion of the correlated mutation information increases of 5 percentage points the SVR performance (from 54% to 59%). When this approach is used in combination with a method previously developed by us and scoring at the state of art in predicting both location and topology of disulfide bonds in Eukaryotes (DisLocate), the per-protein accuracy is 38%, 2 percentage points higher than that previously obtained.

**Conclusions:**

In this paper we show that the inclusion of information derived from correlated mutations can improve the performance of the state of the art methods for predicting disulfide connectivity patterns in Eukaryotic proteins. Our analysis also provides support to the notion that improving methods to extract evolutionary information from multiple sequence alignments greatly contributes to the scoring performance of predictors suited to detect relevant features from protein chains.

## Background

Disulfide bonds are covalent cross-links between cysteine side chains that play very important roles in the native structures of globular proteins. Folding, stability, and ultimately function of secreted proteins in cells are influenced by the formation of disulfide bonds between cysteine residues [1]. Predicting the topology and the location of disulfide bridges in a protein from its sequence therefore plays a relevant role in protein structural and functional annotation. Several computational methods are presently available for computing cysteine properties in a protein sequence and they can be grouped into: i) methods that predict the disulfide bonding state [2-4]; ii) methods that predict the topological connectivity patterns by assuming that the cysteine bonding state is known [5-8]; iii) methods that compute both i) and ii)[9-12]. Recently we developed DisLocate, a two-stage method for disulfide bond prediction in Eukaryotes comprising two integrated modules. The first based on Conditional Random Fields (CRFs) predicts the cysteine bonding state; the second based on a Support Vector Regression (SVR) predicts the topology of the disulfide bridges [12]. DisLocate improved over previous methods by introducing for the first time the information of the protein subcellular localization in the prediction of the disulfide bonding state [12].

Here we address the problem of improving the second step of the prediction, namely the prediction of disulfide connectivity pattern, by exploiting the role of correlated mutations. Correlated mutation analysis aims at elucidating relations between pairs of residues in the protein structure that may influence its folding. Routinely, this is done through the identification of the co-evolution of different positions in a multiple sequence alignment. The notion of correlated mutation describes that an unfavourable residue mutation in a structural contact can be compensated by the simultaneous change of the direct partner in such a way that the original interaction is preserved (compensatory mutation) [13]. It has been recently observed that with sufficient and correct information about protein residue-residue contacts it is possible to predict some protein structures from the residue chain [13-16].

Correlated mutation analysis was also introduced in the context of disulfide bond connectivity prediction. Simple correlation patterns of concerted appearing and disappearing cysteines in multiple structural alignments were used to predict the topology of disulfide bonds in proteins [17].

In the present paper we propose the usage of information derived from correlated mutations to improve the prediction of disulfide connectivity over a set proteins including 1797 chains (PDBCYS). We evaluate two different approaches of computing the correlated mutations: corrected mutual information (MIp) and sparse inverse covariance estimation (iCOV). MIp is a corrected version of mutual information specifically designed to remove the background noise due to both phylogenetic and entropic biases [18]. The latter approach (iCOV) which is based on sparse inverse covariance estimation was recently introduced for the problem of predicting contact maps [20]. Here we combine information derived with both methods for computing correlated mutations with features that were previously found relevant for predicting the disulfide connectivity and implemented in our DisLocate [12]. In order to highlight the effect of correlated mutations we benchmark the newly developed predictors on the same dataset (PDBCYS) previously adopted to evaluate DisLocate [12]. Our results show that correlated mutation analysis adds to the previously introduced features and improves the prediction scores. This indicates that correlated mutations are a significant piece of information also when computing the connectivity pattern of disulfide bridges in protein structures.

## Methods

### Mutual information among cysteines

Mutual Information (MI) can be used to provide a measure of the co-evolution of two positions in a protein sequence. In protein structures the measures of co-evolution and MI in particular have been extensively applied for predicting residue contacts in proteins [13-16]. Here we focus only on sequence positions that contain cysteine residues. We then compute a multiple sequence alignment for each protein of interest and we extract the positions that correspond to cysteines in the query sequences. By this, we end up with sub-alignments that contain as many columns as the number of cysteines that are present in the query sequence. The Mutual Information MI between cysteines *i *and *j *is then computed as follows:

(1) $$MI\left(i,j\right)=\underset{a,b}{\sum }f_{i,j}\left(a,b\right)\text{log}\frac{f_{i,j}\left(a,b\right)}{f_{i}\left(a\right)f_{j}\left(b\right)}$$

where *f_i_(a) *and *f_j_(b) *are the relative frequencies of amino acid types *a *and *b *at position *i *and *j*, respectively, and *f_i,j_(a,b) *is the relative frequency of the amino acid pair *ab *at positions *ij*.

The MI metric suffers of several drawbacks mainly due to entropic effects and phylogenetic biases that reduce its efficacy in predicting residue contacts [18]. The entropic bias occurs when a given position in the multiple alignment exhibits a high variability (entropy). These positions tend to have higher level of MI than those with a lesser entropy [18]. The phylogenetic bias is due to the phylogenetic relationships between organisms represented in the alignment that may generate an uneven distribution of sequence residues [18]. In order to overcome these issues, it has been proposed to correct the MI values as computed in Equation 1 by the so called *average product correction *(APC) [18]. APC measures the background signal of MI due to entropic and phylogenetic biases. This corrected metric is called MIp. The MIp for positions *i *and *j *is then obtained as follows:

(2) $$MI_{p}\left(i,j\right)=MI\left(i,j\right)-\frac{\bar{MI}\left(i,-\right)\bar{MI}\left(-,j\right)}{\bar{MI}}$$

where $\bar{MI}\left(i,-\right)$ is the average mutual information between position *i *and all other positions (analogously $\bar{MI}\left(-,j\right)$ for position *j*) and $\bar{MI}$ is the average mutual information of all positions.

### Sparse inverse covariance estimation

In recent works it has been pointed out that it is possible to improve the co-evolutionary information using the inverse of the covariance matrix [19,20]. In particular, using information stored into the inverse of the covariance matrix, the performance of contact prediction improves significantly with respect to the simple MI or MIp by reducing the so-called *indirect coupling effect *i.e. the statistical dependency observed in multiple sequence alignment for residues that are structurally distant [19-21]. One of the proposed approach (followed here), is based on sparse inverse covariance estimation and it is called PSICOV [20].

In this paper, we apply an approach similar to PSICOV to estimate the level of direct coupling between cysteine residues in proteins. As a result, given a protein with *m *cysteines, we obtained a *m *by *m *matrix whose elements can be interpreted as disulfide bonding scores. As described for MI in the previous section, we consider multiple sequence alignment constrained to positions corresponding only to cysteines in the query sequence. For a protein sequence with *m *cysteines, the sample covariance matrix can be then computed as follows:

(3) $$S_{i,j}^{a,b}=f_{i,j}\left(a,b\right)-f_{i}\left(a\right)f_{j}\left(b\right)$$

where *f_i_(a), f_j_(b) *and *f_i,j_(a,b) *are defined as in the previous section and *S *is a 21*m *by 21*m *covariance matrix (here we also include the gap as a 21st symbol).

Assuming that the covariance matrix can be inverted (the matrix is not singular), the inverse matrix provides information about the degree of *direct coupling *between different positions in the protein sequence [19,20]. Unfortunately, the covariance matrix can be singular (since we do not observe every amino acid in a given position of the alignment). In order to estimate the inverse matrix, authors proposed to use the sparse inverse covariance estimation by means of the *graphical Lasso *optimization procedure[22,23]. This procedure attempts to estimate the inverse covariance matrix by solving the following optimization problem:

(4) $$\overset{d}{\underset{i,j=1}{\sum }}S_{ij}\Theta_{ij}-\text{logdet}\Theta +\rho \overset{d}{\underset{i,j=1}{\sum }}\left|\Theta_{ij}\right|$$

where *S *is a *d*x*d *covariance matrix, *Θ *is the inverse covariance matrix and the last term is a regularization term (the ℓ_1_-norm of the inverse matrix) that favors the sparsity of the solutions. *ρ *is a hyper-parameter that governs the level of desired sparsity (the greater is *ρ *the sparser is the solution). The disulfide bonding score between cysteines *i *and *j *of the protein is computed as follows:

(5) $$C\left(i,j\right)=\underset{a,b}{\sum }\left|\Theta_{ij}^{ab}\right|$$

where the summation over *a *and *b *is taken by excluding gaps. Finally, the same average product correction is used here to adjust the value for background noise as described for MIp:

(6) $$C_{p}\left(i,j\right)=C\left(i,j\right)-\frac{\bar{C}\left(i,-\right)\bar{C}\left(-,j\right)}{\bar{C}}$$

where $\bar{C}\left(i,-\right)$ is the mean contact score between position *i *and all other positions (analogously $\bar{C}\left(-,\;j\right)$ for position *j*) and $\bar{C}$ is the overall mean contact score. We refer to this bonding score as iCOV in the rest of the paper.

### Predicting disulfide connectivity patterns

Once the cysteine bonding state is assigned, we predict the connectivity pattern of the subsets of proteins that contain at least a pair of cysteines in the bonding state by applying a Support Vector Regression approach [12]. The SVR predictions of each possible pair of cysteines is used as edge weight and the Edmond-Gabow algorithm is adopted to predict the most probable disulfide pattern [5]. In order to evaluate SVR, we use the same 20-fold cross validation procedure described before [12], considering only proteins with at least two disulfide bridges. SVRs were trained using an input encoding based on global and local information. The global information (that does not depend on each particular cysteine pair) is defined by the Normalized Protein Length (one real value), the Protein Molecular Weight (one real value) and the protein amino acid composition (20 real values). The local pairwise encoding (that depends on each particular cysteine pair) consists of the following descriptors:

• two PSSM-based windows centered on the cysteines forming the pairs. We used a window of length 13, the best performing among the different-sized windows we tested. With this choice, we ended up with a vector of 13 * 20 * 2 = 520 components;

• the Relative Order of the Cysteines. This feature is encoded with 2 real values that represent the normalized relative order of a cysteines pair. Given a protein with n cysteines (C1,C2,...,Cn), the corresponding normalized ordered list of cysteines is given by (1/n, 2/n,...,n/n). For each pair of cysteines, the corresponding values are then taken from the list (e.g. the pair (C1,C4) is encoded as (1/n,4/n));

• the Cysteine Separation Distance. This feature is encoded with 1 real value that represents the log-cysteine sequence separation computed as SEP(Ci,Cj) = log (|j - i|) where i and j are sequence positions of cysteines Ci and Cj, respectively.

• Correlated mutation information, based on MIp and/or iCOV.

### Dataset description

In this study we used the dataset PDBCYS introduced before [12]. From PDB (release May 2010) we extracted 1797 Eukaryotic protein structures with resolution <2.5 Å with at least two cysteine residues and global pairwise sequence similarity <25%. PDBCYS includes 7619 free and 3194 bonded cysteines. Since PDBCYS contains pairs of proteins with detectable local sequence similarity, we clustered all the chains using a local sequence similarity score. First, we ran a BLAST sequence search using all the proteins of the set versus themselves. Then, for each pair of proteins we selected the higher bi-directional (say p1 vs p2 or p2 vs p1) sequence identity as reported in the BLAST output. We subsequently treated the proteins as nodes of a graph and assigned an edge between two nodes only where local sequence identity between the corresponding protein sequences was > 25%. In addition, we computed the connected components of the graph and treated each group of nodes as a protein cluster. Finally, the clusters were grouped in 20 disjoint sets used to train and test the method. We used these 20 subsets to evaluate our method and to compare its performance with previous approaches by adopting a 20-fold cross-validation procedure.

### Performance measures

In the following *N_c _*is the number of correctly predicted bonds, *N_p _*is the total number of predicted bonds, *N_b _*is the total number of observed bonds, *N_patt _*is number of correctly predicted disulfide connectivity pattern and *N *is the total number of chains.

To score the disulfide connectivity prediction we computed the following indices:

• the precision *P_b_*:

(7) $$P_{b}=\frac{N_{c}}{N_{p}}$$

• the recall *R_b_*:

(8) $$R_{b}=\frac{N_{c}}{N_{b}}$$

• the *Q_p_*:

(9) $$P_{b}=\frac{N_{patt}}{N}$$

For sake of readability in the Tables we report the indices in percentage (i.e. the obtained values are multiplied by 100).

### Technical details

All multiple sequence alignments used to compute both the MIp and the iCOV features have been generated by running 3 iterations of the jackhmmer program which is a part of the HMMER 3.0 package (http://hmmer.org) against the UNIREF90 sequence database. The inverse covariance estimation was performed by means of the *glasso *R package available at the CRAN archive (http://cran.rproject.org/web/packages/glasso/index.html), the same used in [12]. All the estimations have been performed using the exact algorithm of the *glasso *code (see *glasso *package documentation for details). *glasso *algorithm depends on a parameter ρ that conditions the sparsity of the reconstructed inverse covariance matrix. This parameter also affects the algorithm run time: the smaller is ρ the longer is the required time. Below we report the results obtained when ρ is set to 1e-8, that was chosen as trade-off between the computational time and the method performance (computed on the validation sets). MIp values were computed as described in [18]. For the SVR implementation we used the libsvm package (http://www.csie.ntu.edu.tw/~cjlin/libsvm/) with a RBF kernel.

## Results and discussion

### Prediction of disulfide connectivity with known bonding state

In order to evaluate the effect of correlated mutations in the task of predicting the topology of disulfide bonds, we first assume that disulfide bonded cysteines are known. We evaluated the performance of methods considering subsets of proteins with a different number of disulfide bonds (from 2 to 5). The reported accuracy was obtained using the same 20-fold cross validation procedure previously described [12].

In Table 1 the results obtained by evaluating only the correlated mutation information are listed. Both MIp and iCOV are evaluated as *unsupervised predictors*. This was done by considering the correlated mutation values computed with the MIp and iCOV algorithms as a measure of the extent of the interaction between pair of cysteines without applying any supervised learning procedure. We constructed two simple predictors by directly interpreting MIp and iCOV as disulfide bonding *potentials *among cysteines and predicting the highest scoring set of cysteine pairs as the most probable disulfide connectivity pattern. The pattern selection was done by computing the maximum-weight perfect matching with the Edmond-Gabow algorithm as previously described [5]. The performance of these unsupervised predictors reported in Table 1 (43.7-44-5% of Qp and 49.9-51.7% of Pb), are significantly higher than a random predictor and higher than methods that do not include evolutionary information [5]. Differently from the case of contact prediction [20], in our case MIp routinely outperformed iCOV with the exception of the case of three disulfide bonds, where iCOV obtained the highest score.

**Table 1:** Performance on disulfide connectivity prediction obtained with correlated mutation measures

# bonds	ICOV		MIp		Random	
	Pb = Rb	Qp	Pb = Rb	Qp	Pb = Rb	Qp
**2**	62	62	68	68	33	33
**3**	52.6	42.4	47.8	37.7	20	7
**4**	51.8	26.8	49.4	29.3	14	1
**5**	39.5	16.2	33.5	13.5	11	0.1
**All**	51.7	43.7	49.9	44.5	23	15

**#bonds**: number of disulfide bonds; iCOV: sparse inverse COVariance estimation; MIp: MIp: corrected Mutual Information. Random: performance obtained by a random predictor. Here Pb = Rb since the total number of predicted bonds (which is known in this experiment) is equal to the total number of observed bonds (*N_p _*= *N_b_*). For index definition see Performance measures.

In Table 2 we report the performance of the SVR-based predictors that include in their input the correlated mutation information. For sake of comparison, we also report the accuracy per protein of the best method based on SVR that does not take advantage of the correlated mutation information but exploits all the other input features described in the Method section (Table 2, under the column labelled SVR). SVR is equivalent to the second module of DisLocate [12]. From the data listed in Table 2, it is evident that when the correlated mutation information is included in the SVR input, the overall performance increases (compare SVR with SVR+iCOV and SVR+MIp). In both cases they outperform the baseline SVR predictor, in particular for proteins that contain 4 and 5 disulfide bonds (it was previously discussed that the difficulty of the prediction increases as the number of cysteines increases[5]). It is also worth noticing that iCOV seems to add more information with respect to MIp as indicated by the relative scoring values (columns SVR+iCOV and SVR+MIp). Since iCOV and MIp appear to capture different aspects of the correlation between cysteines, we also evaluated a SVR-based predictor that includes among its features both correlation measures (SVR+iCOV+MIp). In this case the performance of the SVR further increases and overpasses by 5 percentage points the recently published SVR method (second step of DisLocate in [12]).

**Table 2:** Performance on disulfide connectivity prediction obtained with different SVR-based methods

# bonds	SVR		SVR+iCOV		SVR+MI		SVR+MI+iCOV	
	Pb = Rb	Qp	Pb = Rb	Qp	Pb = Rb	Qp	Pb = Rb	Qp
**2**	75	75	76	76	73	73	76	76
**3**	60	48	62.8	55.3	59.6	50.6	62.8	55.3
**4**	57	44	67.1	51.2	61	46.3	67.7	51.2
**5**	46	19	55.1	27	54.1	29.7	58.9	32.4
**All**	60	54	65.2	58.6	61.9	55.5	66.2	59.3

**# bonds**: number of disulfide bonds; MIp: corrected Mutual Information; iCOV: sparse inverse COVariance estimation; SVR: Support Vector Regression; and their combinations as indicated. For details see Methods. Results are evaluated on the PDBCYS dataset [12]. SVR results are taken from [12]. For index definition see Performance measures.

### Prediction of disulfide connectivity with predicted bonding state

In real cases, when new protein sequences are analysed, it is not known if some of the cysteines are making disulfide bonds in the three dimensional structure of the protein. It is then useful evaluating the predictor accuracy starting from an unlabelled sequence and predicting both the disulfide bonding states and also the connectivity pattern. We evaluated the performance of the connectivity pattern predictor based on SVR+iCOV+MIp when the bonding state of cysteines is not known but *it is predicted*. For this purpose, we adopted the bonding state predictor previously introduced in DisLocate which is based on Grammatical-Restrained Hidden Conditional Random Fields [24] and protein subcellular localization [12]. In this case, we used the GRHCRF part of DisLocate to predict the bonding state and the new predictor to assign the connectivity pattern. For sake of comparison, we evaluate the method adopting the same experimental setup previously described and using the same cross-validation procedure [12]. Results are shown in Table 3 and indicate that the improvement over DisLocate is 2 percentage points of accuracy per protein.

**Table 3:** Prediction without a prior knowledge of the cysteine bonding state

# bonds	DisLocate			SVR+MI+iCOV		
	Rb	Pb	Qp	Rb	Pb	Qp
**1**	83	46	76	93	46	76
**2**	67	52	61	71	59	62
**3**	47	41	35	55	49	38
**4**	52	37	35	63	48	38
**5**	39	39	15	50	49	16
**All**	52	42	36	60	50	38

Legends are as in Table 2.

### Prediction performance as a function of the quality of the multiple sequence alignments

MIp and iCOV are computed over multiple sequence alignments. We therefore evaluate how the number and the type of sequences included in the alignment (used to compute the correlation among cysteines residues) can affect the final result.

**Figure 1:**
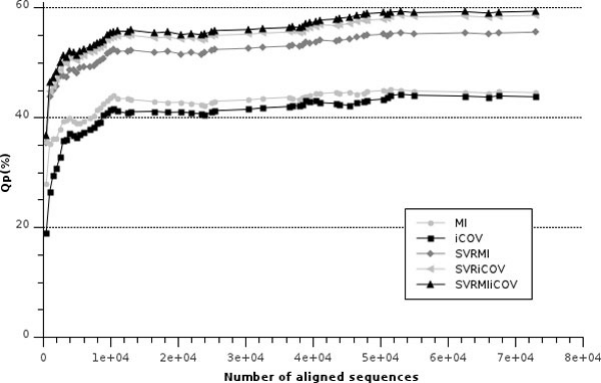
**Scoring the method at increasing number of sequences in the MSA**. The accuracy per protein (Qp) of the different methods is plotted as a function of the number of protein chains in the multiple sequence alignment (*MSA quality*) used to derive information on correlated mutations. MIp: corrected Mutual Information; iCOV: sparse inverse COVariance estimation; SVR: Support Vector Regression; and their combinations as indicated. For details see Methods.

**Figure 2:**
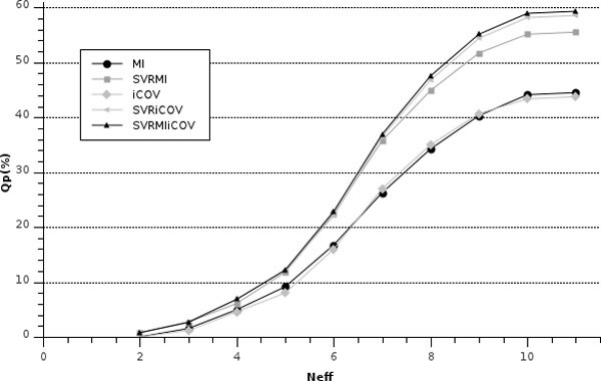
**Scoring the method at increasing NEFF value**. The accuracy per protein (Qp) of the different methods is plotted as a function of the NEFF value (NEFF = 1 single sequence, NEFF = 20 random) [25]. MIp: corrected Mutual Information; iCOV: sparse inverse COVariance estimation; SVR: Support Vector Regression; and their combinations as indicated. For details see Methods.

The number of aligned sequences in each multiple alignment can vary from sequence to sequence. We evaluate the dependence of the method performance on the number of sequences by computing Qp at increasing threshold value of the number of proteins included in the multiple sequence alignment. The results are reported in Figure 1, where it is evident that the method has on average a lower performance on proteins, whose corresponding multiple sequence alignments contain ≤5000 sequences. Alternatively, when the number of aligned sequences is larger than 10000, the method on average optimally scores. However, a large number of aligned sequences may be not sufficient if the observed sequence variation is not adding any information. In order to highlight this effect, we evaluated the method performance as a function of the number of effective sequences in the alignment (NEFF score) [25]. NEFF is calculated as the exponential of the entropy value averaged over all columns of the multiple-alignment: in this respect NEFF is also interpreted as the entropy of a sequence profile derived from the multiple-alignment [25]. NEFF is a real value ranging from 1 to 20. Multiple sequence alignments consisting of very similar sequences (or singletons) have a NEFF value close to 1, while random (uniform) alignments generate a NEFF of 20. Figure 2 shows that also for the problem at hand, the larger the NEFF value the higher is the method performance, achieving the maximum at NEFF = 10 (in our dataset the maximum value is 11). These findings are in agreement with the notion that the more representative is the multiple sequence alignment, both in terms of sequence abundance and diversity, the higher is the expected predictive performance of the method [19,20].

## Conclusions

The prediction of protein structures from their sequences it is still an open problem in Structural Bioinformatics, especially considering that the disproportion between the number of putative protein sequences with respect to the number of known 3D structures is exponentially increasing. The bonding state of cysteines plays a relevant role in stabilizing the tertiary folds of proteins, in defining protein functions and in triggering functionally relevant conformational changes [26]. The knowledge of disulfide bonds is very important to predict the protein structure in *ab initio *and comparative modelling since it poses constraints to the possible chain conformations [27,28]. In this paper we introduce a new method to predict disulfide bonds starting from protein sequence. We investigate the effect of the information derived from correlated mutations on the problem of predicting the topology of disulfide bonds in proteins. We show that correlated mutations in the form of corrected mutual information (MIp) and inverse of covariance matrix (iCOV) carry a significant quantity of information that was not completely exploited before for the task of disulfide bond prediction. We present a new method that implementing information derived from correlated mutations improves the performance over the state of the art method DisLocate [12]. Finally, we highlight that the optimal performance of the method can be achieved when the number of sequences included in the multiple alignment from where information on correlated mutation is derived is in the range of 10000 protein chains and the correspondent NEFF value of the alignment is greater or equal to 10.

## Authors' contributions

CS carried out all the calculations. CS, PF, RC and PLM conceived the study, analyzed the data and wrote the manuscript. All the authors have read and approved the final manuscript.

## Competing interests

The authors declare that there are no competing interests.

## Declarations

The publication costs of this article were funded by the RFO (Ricerca Fondamentale Orientata) 2011, University of Bologna, awarded to Rita Casadio.

This article has been published as part of *BMC Bioinformatics *Volume 14 Supplement 1, 2013: Computational Intelligence in Bioinformatics and Biostatistics: new trends from the CIBB conference series. The full contents of the supplement are available online at http://www.biomedcentral.com/bmcbioinformatics/supplements/14/S1.
